# Genetically Confirmed Localized Perianal Hailey-Hailey Disease Successfully Treated With Topical Difamilast: A Case Report

**DOI:** 10.7759/cureus.107226

**Published:** 2026-04-17

**Authors:** Maho Maekawa, Kenji Yoshida, Keiji Tanese, Fumiko Ohara, Akira Ishiko

**Affiliations:** 1 First Department of Dermatology, Toho University School of Medicine, Tokyo, JPN

**Keywords:** atp2c1 mutation, difamilast, familial benign chronic pemphigus, hailey-hailey disease, perianal lesion, phosphodiesterase-4 inhibitor

## Abstract

Hailey-Hailey disease, also known as familial benign chronic pemphigus, is a rare autosomal dominant acantholytic dermatosis caused by abnormalities in ATP2C1. The disease typically affects intertriginous areas such as the axillae and groin, whereas localized involvement limited to the perianal region is exceptionally rare. We report the case of a 48-year-old woman who presented with pruritus and pain in the perianal region for one year. The lesion had been refractory to topical corticosteroid therapy. Physical examination revealed a brownish, thickened plaque with erosion and maceration around the anus. Histopathological examination demonstrated suprabasal clefting and acantholysis, creating a dilapidated brick wall appearance. Direct immunofluorescence staining was negative. Genetic analysis identified a donor splice-site mutation in ATP2C1 (c.899+1G>T), confirming the diagnosis of Hailey-Hailey disease. Treatment with topical difamilast, a phosphodiesterase-4 inhibitor, led to improvement of pruritus and gradual epithelialization of the erosion over two months, with no recurrence during seven months of follow-up. Localized perianal Hailey-Hailey disease is an important diagnostic consideration in chronic treatment-resistant perianal erosions. This case highlights the value of careful clinicopathological evaluation and genetic testing in atypical presentations and suggests that topical difamilast may be a useful therapeutic option for localized disease.

## Introduction

Hailey-Hailey disease (HHD), also known as familial benign chronic pemphigus, is a rare autosomal dominant acantholytic dermatosis caused by mutations in ATP2C1 [1-3]. The disease usually presents with recurrent erosive or vegetating plaques in intertriginous areas, such as the axillae, groin, neck, and inframammary regions, where friction, sweating, and secondary infection can exacerbate symptoms [4]. Histopathologically, HHD is characterized by suprabasal clefting and acantholysis, producing the classic dilapidated brick wall appearance [4].

Although the clinical and histological features of classic HHD are well recognized, the mechanisms underlying localized or segmental manifestations remain incompletely understood, and both genetic mosaicism and local environmental triggers have been proposed [5]. Localized involvement limited to the perianal region appears to be exceedingly rare, and only a very small number of such cases have been described in the literature [6-10]. Here, we report a rare case of genetically confirmed localized perianal HHD, highlighting the diagnostic challenge of this unusual presentation and the favorable response to topical difamilast.

## Case presentation

A 48-year-old woman presented with a perineal skin eruption following a one-year history of pruritus and pain in the perineal region.

She had previously been treated with topical hydrocortisone butyrate, a medium-potency corticosteroid, at a local clinic; however, her symptoms showed minimal improvement. Two months before presentation, her pruritus worsened, and she was referred for further evaluation and treatment.

**Figure 1:**
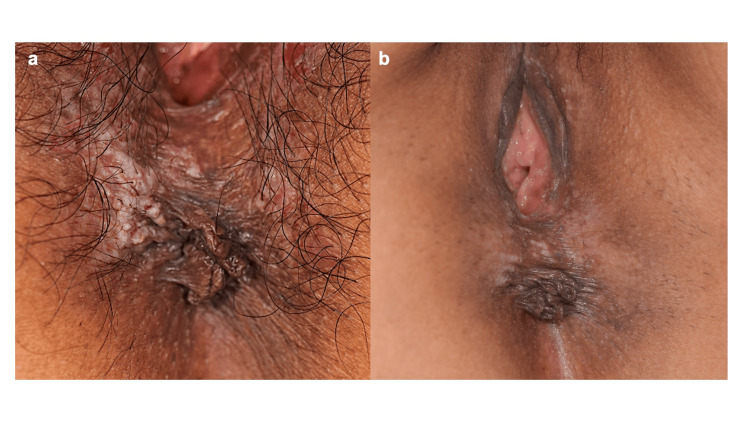
Clinical findings of the perianal lesion. (a) At presentation, a brownish plaque with epidermal thickening was observed around the perianal region, with whitish maceration on the right side. Multiple light-brown flat papules were scattered on the inner aspect of the labia majora, and a small bean-sized superficial erosion was present on the left side of the perineum. (b) After treatment with topical difamilast, the erosions and maceration markedly improved, leaving only mild residual pigmentation.

Physical examination revealed a brownish, thickened plaque with erosion and maceration around the anus. A whitish macerated area was present in the right perianal region. In addition, a light brown flat papule was observed on the inner side of the labia majora, and a small bean-sized erosion was present on the left side of the perineum (Figure 1a).

No lesions were observed in other intertriginous areas, including the axillae, groin, and neck. Laboratory tests showed no significant abnormalities.

**Figure 2:**
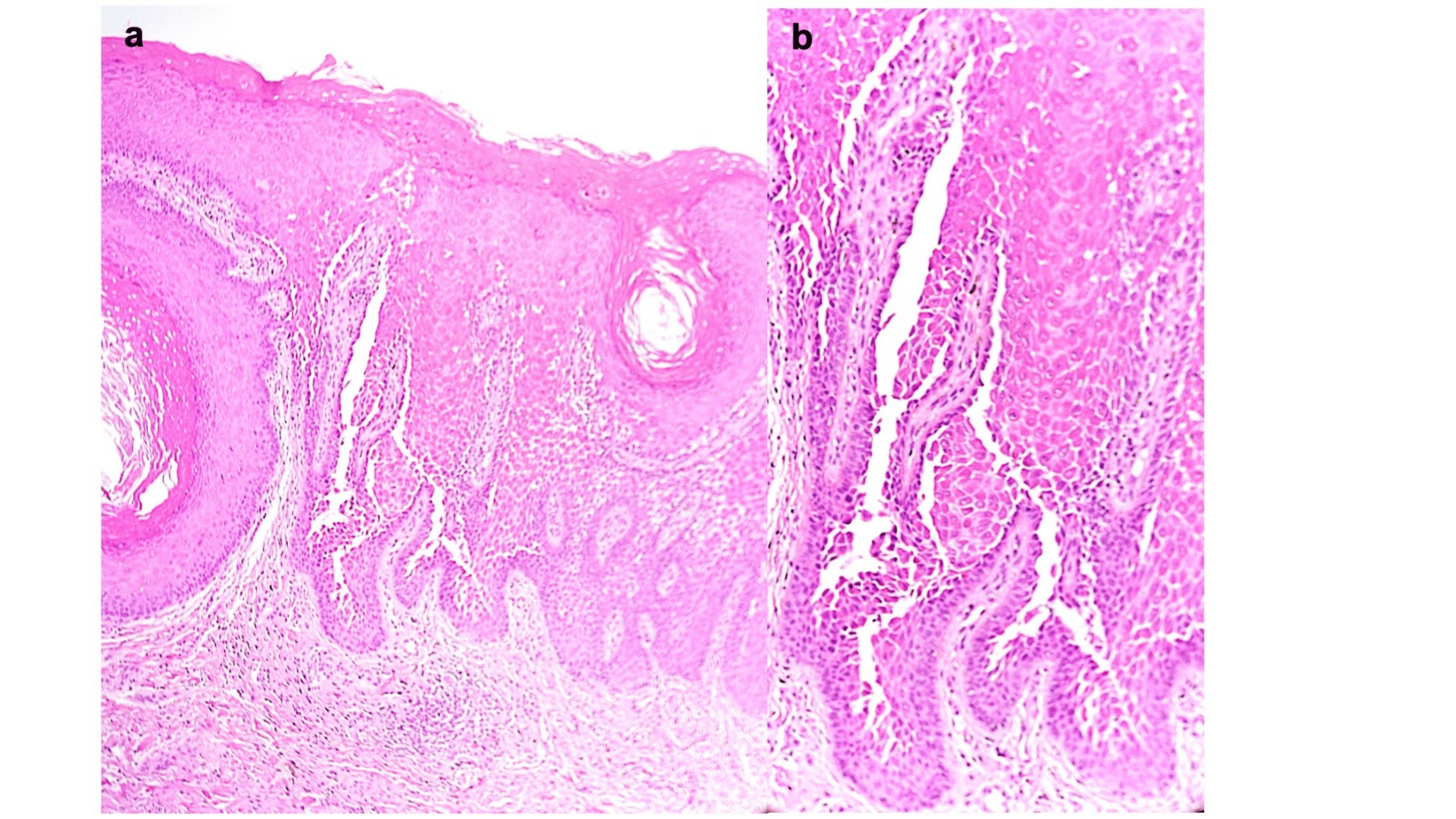
Histopathological findings. (a) Low-power view showing hyperkeratosis and epidermal hyperplasia with suprabasal cleft formation and inflammatory cell infiltration in the superficial dermis. (b) High-power view showing suprabasal clefts with a single layer of basal cells remaining, consistent with the characteristic dilapidated brick wall appearance. No dysplasia was identified.

Histopathological examination of a skin biopsy specimen obtained from the macerated perianal lesion showed hyperkeratosis and acanthosis with suprabasal cleft formation (Figure 2a). Acantholysis above the basal layer created the characteristic dilapidated brick wall appearance of HHD. Mild perivascular lymphocytic infiltration was present in the superficial dermis (Figure 2b). No dysplasia was identified, arguing against a neoplastic process.

Direct immunofluorescence staining was negative, which helped exclude autoimmune blistering disorders. Genetic analysis identified a previously reported donor splice-site mutation in ATP2C1 (c.899+1G>T), confirming the diagnosis of HHD. Because this variant affects a canonical splice donor site, it is predicted to disrupt normal RNA splicing and impair SPCA1 function. Although ATP2C1 mutations are causative for HHD, a clear genotype-phenotype correlation has not been established.

Topical difamilast ointment, a phosphodiesterase-4 (PDE4) inhibitor originally developed for atopic dermatitis, was initiated. By inhibiting PDE4, difamilast increases intracellular cyclic AMP levels and suppresses proinflammatory cytokine production, thereby exerting anti-inflammatory effects. Following treatment, pruritus improved, and gradual epithelialization of the erosion was observed over two months. At six months after treatment initiation, marked epithelialization was observed clinically (Figure 1b). No recurrence was observed during the seven-month follow-up period.

## Discussion

The ATP2C1 gene, responsible for HHD, encodes a Ca²⁺/Mn²⁺ ATPase located in the Golgi apparatus, known as secretory pathway Ca²⁺/Mn²⁺-ATPase 1 (SPCA1) [2,3,5]. SPCA1 plays an essential role in maintaining calcium homeostasis within the Golgi apparatus and is involved in the post-translational processing and trafficking of desmosomal proteins such as desmogleins and desmocollins. Impaired SPCA1 function leads to defective desmosome formation, leading to weakened cell-cell adhesion and subsequent epidermal acantholysis [2,3].

Localized HHD confined to the perianal region, as observed in this case, is extremely rare, and only a limited number of cases have been reported in the literature (Table 1) [6-14].

**Table 1:** Summary of previously reported cases of Hailey-Hailey disease localized to the perianal or vulvar regions.

Author	Year	Country	Age/sex	Clinical presentation	Genetic confirmation	Diagnostic delay
Cooper [12]	1971	Not reported	Not reported	Erosions	No	Not reported
Rabinovitz and Patel [13]	1982	Not reported	Not reported	Verrucoid lesions	No	Not reported
Ewald and Gross [6]	2008	Germany	25/F	Papules	No	Not reported
Wang et al. [7]	2019	China	51/M	Plaques	No	Seven months
Hammami et al. [14]	2022	Tunisia	Not reported	Lichenoid lesions with crusted erosions	No	Not reported
Zhu et al. [8]	2024	China	43/F	Isolated lesion	Yes	Three years
Present case	2026	Japan	48/F	Erosion	Yes	One year

Clinically, localized perianal HHD has most commonly presented as erosion, with other reported manifestations including maceration, erythema, papules, plaques, and isolated perianal lesions. Temporary improvement may occur with antifungal agents or topical corticosteroids; however, recurrence is common. Accurate diagnosis of chronic erosive lesions in the perianal region can be challenging because they may mimic a variety of inflammatory, infectious, and neoplastic disorders, including eczema, candidiasis, contact dermatitis, inverse psoriasis, Bowen’s disease, and extramammary Paget’s disease.

In many previously reported cases, patients were initially treated for eczema or infection, and the correct diagnosis was delayed; among cases with explicitly reported disease duration, the interval before correct diagnosis ranged from seven months to three years [7,8]. Helpful differentiating features include a chronic relapsing course, poor response to conventional treatment, negative direct immunofluorescence, and characteristic histopathological findings of suprabasal acantholysis with a dilapidated brick wall appearance.

HHD is known to be exacerbated by local environmental factors such as friction, sweating, and moisture [4]. Two mechanisms have been proposed for the development of localized HHD: one involves somatic mosaic mutations, and the other involves localized induction of lesions by environmental triggers in patients with germline ATP2C1 mutations [9]. The former typically presents with lesions distributed along the lines of Blaschko, whereas the latter occurs when local mechanical or physical stimuli, such as friction, moisture, or heat, induce lesions in anatomically susceptible areas.

Recently, apremilast has been reported to be effective in the treatment of HHD, and topical agents such as tacrolimus have also been used successfully [10,11]. In the present case, an ATP2C1 mutation was identified in peripheral blood despite the absence of lesions elsewhere on the body, suggesting that local environmental factors contributed to the localized manifestation of the disease. Friction, moisture, maceration, and defecation-related irritation in the perianal area may have acted as local triggers. Given that systemic PDE4 inhibitors may cause diarrhea, which could worsen the perianal condition, topical difamilast ointment was selected. Difamilast may have contributed to clinical improvement by suppressing local inflammation and pruritus, thereby reducing scratching-induced mechanical irritation and promoting re-epithelialization. Topical calcineurin inhibitors and systemic PDE4 inhibitors, such as apremilast, have shown benefit in HHD; however, topical difamilast may offer a localized anti-inflammatory approach with less systemic exposure and potentially fewer systemic adverse effects. Treatment resulted in improvement of pruritus and epithelialization of the erosion without recurrence, suggesting that topical PDE4 inhibitors may represent a useful therapeutic option for localized perianal HHD. However, this report is limited by its single-case design and the relatively short follow-up period of seven months. Given the chronic relapsing nature of HHD, longer follow-up is needed to determine the durability of response and long-term recurrence control.

## Conclusions

To our knowledge, this is among the very few genetically confirmed cases of isolated perianal HHD and one of the first reports describing successful treatment with topical difamilast. This case highlights the importance of considering HHD in the differential diagnosis of chronic treatment-resistant perianal erosions and underscores the value of careful clinicopathological evaluation and, when necessary, genetic testing in atypical presentations. Although limited by its single-case design and relatively short follow-up period, this report suggests that topical difamilast may represent a useful localized therapeutic option.
